# Nano-Motion Analysis for Rapid and Label Free Assessing of Cancer Cell Sensitivity to Chemotherapeutics

**DOI:** 10.3390/medicina57050446

**Published:** 2021-05-04

**Authors:** Petar Stupar, Ana Podolski-Renić, Maria Ines Villalba, Miodrag Dragoj, Sofija Jovanović Stojanov, Milica Pešić, Sandor Kasas

**Affiliations:** 1 Laboratory of Biological Electron Microscopy, Ecole Polytechnique Fédérale de Lausanne (EPFL), 1015 Lausanne, Switzerland; p.stupar@outlook.com (P.S.); ines.villalba@epfl.ch (M.I.V.); sandor.kasas@epfl.ch (S.K.); 2 Department of Neurobiology, Institute for Biological Research “Siniša Stanković”—National Institute of Republic of Serbia, University of Belgrade, Despota Stefana 142, 11060 Belgrade, Serbia; ana.podolski@ibiss.bg.ac.rs (A.P.-R.); miodrag.dragoj@ibiss.bg.ac.rs (M.D.); sofija.jovanovic@ibiss.bg.ac.rs (S.J.S.); 3 Centre Universitaire Romand de Médecine Légale, UFAM, Université de Lausanne, 1015 Lausanne, Switzerland

**Keywords:** atomic force microscope, cantilever, nano-motion, cancer cells, multidrug resistance, personalized therapy

## Abstract

*Background and Objectives*: Optimization of chemotherapy is crucial for cancer patients. Timely and costly efficient treatments are emerging due to the increasing incidence of cancer worldwide. Here, we present a methodology of nano-motion analysis that could be developed to serve as a screening tool able to determine the best chemotherapy option for a particular patient within hours. *Materials and Methods*: Three different human cancer cell lines and their multidrug resistant (MDR) counterparts were analyzed with an atomic force microscope (AFM) using tipless cantilevers to adhere the cells and monitor their nano-motions. *Results*: The cells exposed to doxorubicin (DOX) differentially responded due to their sensitivity to this chemotherapeutic. The death of sensitive cells corresponding to the drop in signal variance occurred in less than 2 h after DOX application, while MDR cells continued to move, even showing an increase in signal variance. *Conclusions*: Nano-motion sensing can be developed as a screening tool that will allow simple, inexpensive and quick testing of different chemotherapeutics for each cancer patient. Further investigations on patient-derived tumor cells should confirm the method’s applicability.

## 1. Introduction

Cancer is a multifunctional disease recognized as a major worldwide health problem, affecting men and women in economically developed countries as well as in developing regions [1]. Unfortunately, cancer remains an unsolved health issue despite the significant advances in our understanding of various aspects of its initiation, progression, metastasis and interactions with the immune system and other normal cells in the tumor microenvironment. In addition, the main obstacle for efficient cancer treatment is development of resistance to current treatment options [2].

Resistance can occur prior to drug treatment (primary or innate resistance) or may develop over time after exposure to the drug (acquired resistance) [3]. Treatment with a single chemotherapeutic agent may lead to the development of resistance to multiple structurally and functionally unrelated compounds, known as cross-resistance or multidrug resistance (MDR). MDR frequently correlates with overexpression of the ATP-Binding Cassette (ABC) transporters in cell membranes that actively pump anticancer drugs out of cells [4]. Overexpression of membrane transporter P-glycoprotein (P-gp) is the most common alteration in cancer cells with MDR phenotype [5].

Since cancer is characterized by its pleomorphism and its variations from patient to patient, personalized therapy has emerged nowadays as the most efficient way to fight this incurable disease [6]. In order to collect genetically accurate data and find appropriate personal therapy, researchers cultivate patient-derived primary tumor cells. This allows the detection of intrinsic MDR as well as the identification of therapeutics valuable for particular cancer patient treatment. Besides increasing efficacy, this kind of approach can minimize adverse effects [7]. Unfortunately, the methods currently used for generating and culturing primary tumor cells are time-consuming, expensive and require specialized laboratories and personnel. Therefore, personalized cancer treatment requires major investments in infrastructure and in the development of assays to make it accessible to a wide range of patients [8]. In addition, the studies of drug effects on artificially cultured tumor cells that change during propagation may not reflect the situation *in vivo*. Ideally, a drug profiling tool should be able to perform analyses as a function of drug concentration, and yet be simple, inexpensive and quick enough to allow extensive dose–response profiles for many drugs. Such an innovative and versatile technique would have the potential to greatly facilitate the development of new therapeutics and their combinations, but also contribute to the emerging field of personalized medicine.

Recently we have introduced an innovative technique, called nano-motion detection that could offer an interesting alternative to the existing tests. The technique consists in attaching the sample of interest onto a nanomechanical sensor (atomic force microscope (AFM) cantilever in this case) and in monitoring its fluctuations as a function of different physicochemical stimuli. We demonstrated that the oscillations of the cantilever last as long as the specimen is alive and stop immediately when the organism dies. Importantly, we also highlighted that variance of the oscillation signal of the cantilever reflects the metabolic activity of the living specimens. We have exploited this technique at first as a result of assessing the sensitivity of bacteria to antibiotics [9,10]. Later we extended its application to proteins and different biological systems, including single mammalian and plant cells [11]. The aforementioned works, in particular those involving the study of bacteria and mammalian cells, indicated that this technique can be extended to the field of oncology. As a rapid and single-cell diagnostic tool, nano-motion sensors could facilitate the search for the best chemotherapy regimen for different cancer types, most importantly resistant ones. The final objective is testing of patient tumor tissue samples and obtaining guidelines for personalized treatment. The very first studies involving nano-motion detection of cancer cells were conducted by Kasas et al. [12] and Wu et al. [13] in 2015 and 2016, respectively. These preliminary experiments clearly demonstrated the capacity of this technique to detect rapidly, and in a label free manner, sensitivity or resistance of cancer cells to anticancer drugs.

In this contribution we extended these preliminary studies to six different human cancer cell lines, three sensitive and their three MDR counterparts. MDR cancer cell lines were established by continuous exposure to classical chemotherapeutics, i.e., doxorubicin (DOX) or paclitaxel (PTX). They all overexpress P-gp. Specifically, the cell lines we used originate from non-small cell lung carcinoma (NCI-H460 and NCI-H460/R) [14]; colorectal carcinoma (DLD1 and DLD1-TxR) [15] and glioblastoma multiforme (U87 and U87-TxR) [15]. The aim of our study was to investigate whether this technique is suitable to differentiate quickly and efficiently sensitive from resistant cancer cells. Despite that the technique is single cell sensitive, in this study we preferred to deposit 2–5 cells onto the sensor to increase the signal to noise ratio. Further studies involving single cell experiments with dose response tests will be necessary to assess nano-motion detection as a valuable technique for cancer research or personalized medicine applications.

## 2. Materials and Methods

### 2.1. Drugs and Chemicals

DOX solution was obtained from EBEWE Arzneimittel GmbH, Vienna, Austria and kept at −20 °C. RPMI 1640 medium, minimum essential medium (MEM), fetal bovine serum (FBS), non-essential amino acids, antibiotic–antimycotic solution, penicillin–streptomycin solution, l-glutamine, and trypsin/EDTA were purchased from Bioind, Beit Haemek, Israel.

### 2.2. Cells and Cell Culture

NCI-H460, DLD1, and U87 cell lines were purchased from the American Type Culture Collection, Rockville, MD, USA. NCI-H460/R cells were selected originally from NCI-H460 cells in a medium containing 100 nM DOX [14]. DLD1-TxR and U87-TxR cells were selected by continuous exposure to stepwise increasing concentrations of PTX (100–300 nM) for a period of ten and nine months from DLD1 and U87 cells, respectively [15]. MDR cancer cell lines (NCI-H460/R and DLD1-TxR) and their sensitive counterparts (NCI-H460 and DLD1) were maintained in RPMI 1640 medium supplemented with 10% FBS, 2mM l-glutamine and 10,000 U/mL penicillin, 10 mg/mL streptomycin, and 25 μg/mL amphotericin B solution, while U87 and U87-TxR were grown in MEM with 10% FBS, 1% non-essential amino acids, 2 mM l-glutamine and 5000 U/mL penicillin, and 5 mg/mL streptomycin solution. All cell lines were sub-cultured at 72 h intervals using 0.25% trypsin/EDTA and seeded into a fresh medium at the following densities: 8000 cells/cm2 for NCI-H460, DLD1, and DLD1-TxR, 16,000 for U87 and NCI-H460/R, and 32,000 for U87-TxR.

### 2.3. Assessment of Cell Proliferation in Real-Time

Continuous cell proliferation of NCI-H460 and NCI-H460/R cancer cells untreated or treated with 500 nM DOX was analyzed using the xCELLigence Real Time Cell Analyzer (ACEA Biosciences Inc., Santa Clara, CA, USA) which facilitates label free real-time cell analysis by measuring impedance-based signals across a series of gold electrodes. Using E-plates, 50 µL of complete medium RPMI 1640 was added to each well and the electrodes were allowed to stabilize for 30 min. The plates were then moved into the xCELLigence Real Time Cell Analyzer to set a base line without cells. The cells were then seeded on E-plate at a following density—4000 cells per well. Cells on the electrodes were monitored by reading and recording the cell impedance every 30 min through 165 h.

### 2.4. Nanomotion Sensor Preparation

For all our experiments, commercial tipless AFM cantilevers (NP-O10 Bruker, Billerica, MA, USA) with nominal spring constant of 0.12 N/m were used. To ensure the adhesion and spread of the cells on the cantilevers, the sensors were chemically functionalized. Prior to introduction into the analysis chamber, they were exposed to 50 μg/mL fibronectin diluted in sterile water for 15 min (Sigma Aldrich, Saint Louis, MO, USA). After this period, they were thoroughly washed with ultrapure water and used for the nano-motion experiments. The sensitivity calibration (i.e., conversion of the photodetector signal in quantitative value of distance/force) was completed by correlating the extension of the piezoelectric crystal of the scanner with the deflection of the laser beam on the photodiode while the cantilever was kept in contact with a hard surface (glass in our case). The determination of the cantilever spring constant was performed by using the “thermal noise method”. Both calibration procedures are implemented in the JPK operating software (“thermal noise method”).

### 2.5. Experimental Procedure

**Figure 1:**
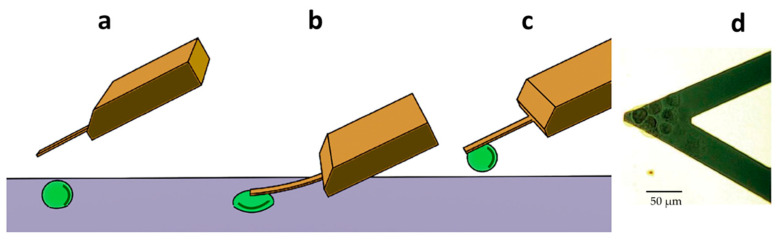
Setup of the nano-motion measurements. (**a**) The AFM cantilever is placed above a living cell. (**b**) The cantilever is lowered until it comes in contact with the cell to promote its attachment onto the lever. (**c**) The cantilever and the cell are raised above the surface to start the cantilever oscillation measurements. (**d**) Optical microscopy image of the cantilever loaded with several cells.

For all experiments, a Nanowizard III AFM from JPK Instruments (Berlin, Germany) accompanied with a Zeiss Axiovert inverted optical microscope was used. We produced a PDMS custom analysis chamber [16], which was then placed in the optical line of the microscope. At this point, 3 mL of complete culture medium was introduced in the chamber as well as a small quantity (30–50 µL) of buffer with a cellular concentration adjusted to reach the number of 5–10 cells in the field of view of the microscope after sedimentation. The cells were let to sediment on the bottom of the analysis chamber and a 5 min measurement of the fluctuations of the sensor in the medium without cells was performed. After this stabilization period, we immobilized some cells on the surface of the cantilever by cell “fishing”, described in detail elsewhere [12,17,18,19]. Specifically, we used the AFM coarse and fine movement capabilities to approach a single cell, attaching it near the apical region of the cantilever. The attachment consisted in placing the cantilever on top of a single cell and lowering it to exert a constant force between 1–5 nN for at least 3 min. Typically, we chose to attach 2–5 single cells to the sensor, ensuring that all cells stayed on the apical region of the cantilever (Figure 1). After the attachment phase, we gradually retracted the sensor to avoid interference from the substrate and the cells were left for 1 h on the cantilever to spread. During this period, we continuously measured, at 10 kHz acquisition frequency, the fluctuations of the cantilever to determine the healthy state of the cells before exposure to the drugs. After the stabilization period, which was not constant in every experiment (15–45 min), we flushed the analysis chamber with the chosen concentration of DOX and followed the resulting response of the cells both by nano-motion analyses and using conventional optical microscopy. We used a ProgRes MF cool, Jenoptik ProgRes^®^ camera (Jena, Germany) connected to the inverted microscope to observe the cells on the sensor every 20 s.

### 2.6. Statistical Analysis

The statistical analyses were performed by Student’s *t*-test and Mann-Whitney non-parametric *t*-tests. Difference was considered to be statistically significant if *p* < 0.05.

## 3. Results and Discussion

In order to investigate whether the nano-motion sensor could be used to identify cancer cells which are resistant to chemotherapy, we employed an in vitro MDR model system that comprises three different sensitive human cancer cell lines and their MDR counterparts. P-gp that is overexpressed in tested MDR cancer cell lines is a member of the ABC transporter family which reduces intracellular accumulation of anticancer drugs by increasing drug efflux, thus suppressing the interaction between the drug and its intracellular target molecule [20]. P-gp can bind a wide variety of hydrophobic drugs including DOX and PTX [21].

**Figure 2:**
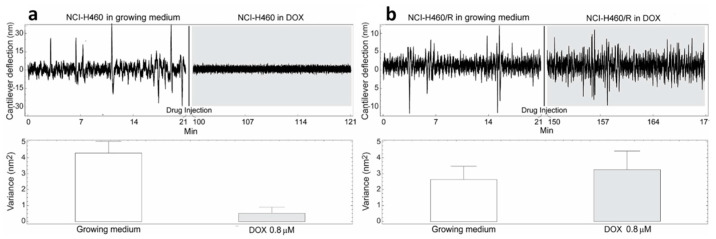
Nanomotion measurements of NCI-H460 (**a**) and NCI-H460/R (**b**) cells before and after exposure to 0.8 µM DOX. Upper panels: typical cantilever deflection signal, lower panels cantilever deflection variance signal. The injection of the drug occurred at 21 min (n = 3 for each experiment).

**Figure 3:**
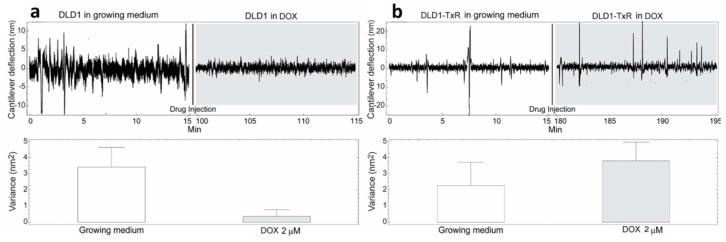
Nanomotion measurements of DLD1 (**a**) and DLD1-TxR (**b**) cells before and after exposure to 2 µM DOX. Upper panels: typical cantilever deflection signal, lower panels: cantilever deflection variance signal. The drug was injected at 15 min (n = 3 for each experiment).

**Figure 4:**
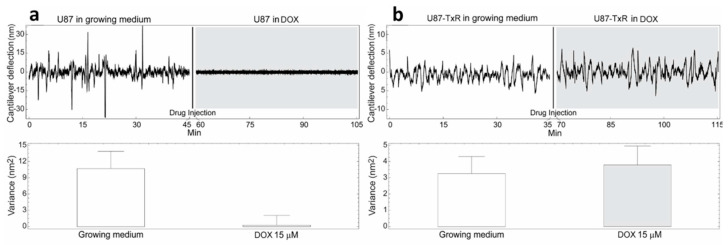
Nanomotion measurements of U87 (**a**) and U87-TxR (**b**) cells before and after exposure to 15 µM DOX. Upper panels: typical cantilever deflection signal, lower panels: cantilever deflection variance signal. The drug was injected in the analysis chamber at 45 (left panel) and 35 (right panel) min. (n = 3 for each experiment).

The cells were immobilized on a cantilever sensor (Figure 1) and cantilever fluctuations were measured. The fluctuations of the sensor produced by the presence of the cells attached to its surface were well pronounced, in a frequency range of 0.1–100 Hz and with amplitude which depends on the position and number of cells, but typically at around 10 nm (Figure 2, Figure 3 and Figure 4).

Our previous studies with bacteria and cells suggest that these movements represent the viability of cells and convey information about their metabolism, cytoskeleton rearrangements, etc. [22,23].

The cells were exposed to different concentrations of DOX depending on their level of resistance to this drug. Glioblastoma cells were treated with the highest DOX concentration—15 µM (Figure 4). The resistance of U87 cells considered as sensitive in our model is probably due to the deregulation of the cell cycle and the absence of apoptosis [24]. The death of tested sensitive cells (NCI-H460, DLD1 and U87) occurred after 80, 85 and 15 min (respectively) of drug injection (Figure 2, Figure 3 and Figure 4), and the corresponding drops in variance were statistically significant (Table 1). The movement of MDR cells (NCI-H460/R, DLD1-TxR and U87-TxR) continued during 150, 180 and 70 min (respectively) after drug injection (Figure 2, Figure 3 and Figure 4), with an increase in variance that was not significant (Table 1).

Comparing the variance of the nano-motion response before and after the exposure to the drug, we could see that in less than 2 h the susceptible cells decrease the fluctuation variance. The overall movement for these cells after this time-period reverts to the basal level measured before the attachment of the cells. On the other hand, the resistant cells maintained their overall movement even several hours after the exposure to the DOX. In fact, the response of MDR cells to drug attack resulted in a slight increase in movement. The best interpretation of these results is that, in the case of susceptible cells, the loss of cell viability caused by the exposure to the drug halts all biological-related movement of the sensor. The resistant counterparts, on the other hand, retained their metabolic activity and viability, and the movement increase could indicate a metabolic response of the cells to the drug pressure. Hence, the methodology presented could be employed for the distinction of resistant from sensitive tumor cells. This is of great importance in the efforts to personalize cancer therapy. Patient-derived primary tumor cells could be tested accordingly and every patient would obtain specific therapy. This would allow not only a treatment with less adverse effects but would also influence the lowering of treatment costs.

**Figure 5:**
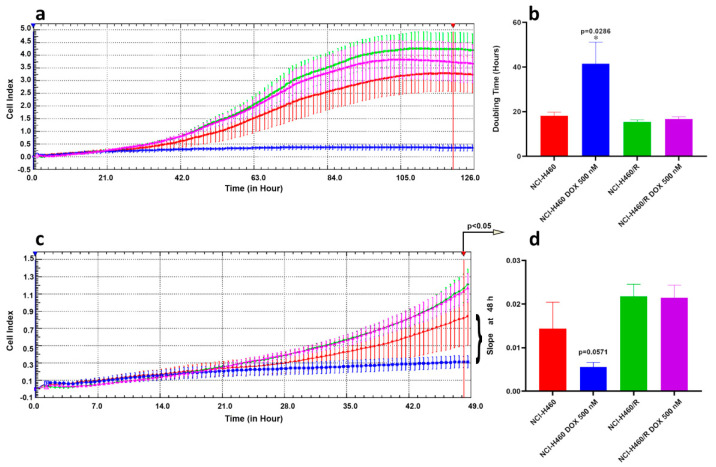
Cell proliferation of non-small cell lung carcinoma cell lines in real-time. (**a**) Continuous cell proliferation of NCI-H460 and NCI-H460/R cells untreated or treated with 500 nM DOX assessed by xCELLigence Real Time Cell Analyzer. X-axes: time in hours; Y-axes: Cell Index that corresponds to impedance measurement. (**b**) Doubling Time (dt) calculated using RTCA 1.2.1 software and analyzed by GraphPad Prism6. Mann-Whitney non-parametric *t*-test revealed statistically significant increase in dt only in NCI-H460 cells treated with 500 nM DOX. (**c**) Illustration of the differences in the slope of the growth curves presented in (**a**). X-axes: time in hours; Y-axes: Cell Index that corresponds to impedance measurement. (**d**) First significant differences in the cell growth can be detected after 48 h, *p* < 0.05 (NCI-H460 untreated cells vs. cells treated with 500 nM DOX, at 48 h *p* = 0.0571 according to Mann-Whitney non-parametric *t*-test).

In addition, by assessment of non-small lung carcinoma cells proliferation in real-time, we confirmed that NCI-H460 cells lose their proliferation ability when treated with 500 nM DOX, while NCI-H460/R treated with the same concentration retain proliferating ability (Figure 5). According to obtained results, doubling time (dt) for untreated NCI-H460 and NCI-H460/R cells and NCI-H460/R cells treated with 500 nM DOX was below 20 h, while estimated dt for NCI-H460 cells treated with 500 nM DOX increased over 40 h showing significant difference with *p* = 0.0286 (Figure 5a,b). Although dt for NCI-H460 cells treated with 500 nM DOX could be calculated, it is obvious that cells completely stopped proliferating (Figure 5a). However, this effect was obvious only after 48 h (Figure 5c,d) even with sophisticated xCELLigence technology. Specifically, the difference in the slope of the growth curve for NCI-H460 cells treated with 500 nM DOX reached *p* = 0.0571 at 48 h. Afterwards, the difference in the slope become significant compared to untreated NCI-H460 cells (*p* < 0.05).

DOX enters the cell via passive diffusion, generally accumulating intracellularly in a concentration that exceeds the extracellular by 10 to 500—fold. Predominantly DOX accumulates in the nuclear compartments [25]. It has been reported that DOX acts by free radical formation and DNA damage via inhibition of topoisomerase II, inducing different cell death pathways depending on the dose. Low dose induces mitotic catastrophe, characterized by the formation of multiple micronuclei and loss of membrane integrity, while high dose induces apoptosis, characterized by reduction of cell volume and apoptotic blabbing [26].

## 4. Conclusions

Our results showed that the variance of the nano-motion signal directly depends on the cancer cells’ sensitivity to DOX. Further investigations will involve single cell studies with the injection of different drug concentrations to ensure linearity of results and explorations on patient derived primary cancer cells. Such tests will provide more specific and accurate data regarding the method applicability. Importantly, nano-motion detection does not only provide on /off results about viability of the tested cells but, as demonstrated by Longo et al. [10] also reflects, online, the cellular metabolic state, at least in bacteria. This is a significant difference to traditional vitality based optical assays. The findings of this study suggest that with the proposed nanomechanical sensing method, researchers and physicians will be able to obtain very fast (only in a few hours) response profile of patient’s tumor cells to different chemotherapeutics, with high temporal resolution and a limited number of cells. To achieve this goal, further developments are required such as the setup of parallel testing devices that are simpler to use than traditional AFMs. We are convinced that our technique can be developed in a screening tool that is simple, inexpensive and quick enough to allow extensive dose-response testing for a number of chemotherapeutics thus enabling treatment optimization for each cancer patient.

## 5. Patents

WO2013054311A1 WIPO (PCT): Nanoscale motion detector. (2013) Inventors: Sandor Kasas, Giovanni Longo, Giovanni Dietler, Bladimir Alonso, Sarduy Livan.

## Figures and Tables

**Table 1:** Variance of the nano-motion signal before and after exposure to DOX of six different human cancer cell lines, three sensitive and their three MDR counterparts.

	NCI-H460			NCI-H460/R		
	**Growing medium**	**DOX 0.8** **µM**	**% Reduction**	**Growing medium**	**DOX 0.8 µM**	**% Increase**
Variance (nm^2^)	4.31	0.50	0.88	2.62	3.25	0.19
Error bar	0.69	0.35		0.88	1.19	
	** *p* = 0.0010			*p* > 0.50		
	DLD1			DLD1-TxR		
	**Growing medium**	**DOX 2** **µM**	**% Reduction**	**Growing medium**	**DOX 2** **µM**	**% Increase**
Variance (nm^2^)	3.40	0.37	0.89	2.25	3.75	0.40
Error bar	1.22	0.38		1.47	1.25	
	* *p* = 0.0177			*p* > 0.05		
	U87			U87-TxR		
	**Growing medium**	**DOX 15** **µM**	**% Reduction**	**Growing medium**	**DOX 15** **µM**	**% Increase**
Variance (nm^2^)	10.65	0.35	0.97	3.25	3.75	0.13
Error bar	3.22	1.52		1.05	1.25	
	** *p* = 0.0074			*p* > 0.05		

*p* values were calculated by using Student’s *t*-test for unpaired samples (n = 3). *, *p* < 0.05, **, *p* < 0.01.

## Data Availability

Data available on request: The data presented in this study are available on request from the corresponding author.
